# Felt presence and its determinants in young adults: results from three independent samples

**DOI:** 10.3389/fpsyt.2024.1442313

**Published:** 2024-09-13

**Authors:** Julian Maciaszek, Adrianna Senczyszyn, Maksymilian Rejek, Tomasz Bielawski, Marta Błoch, Błażej Misiak

**Affiliations:** Department of Psychiatry, Wroclaw Medical University, Wrocław, Poland

**Keywords:** sense of presence, hallucination, delusion, affective symptoms, psychotic disorder

## Abstract

Felt presence (FP) is a phenomenon that might appear in individuals with mental and neurological disorders as well as those without any specific morbidity. Some studies have indicated that FP is closely related to psychotic symptomatology. Yet, the mechanisms underlying its occurrence remain largely unknown. The present study aimed to disentangle as to whether FP is associated with widely known risk factors of psychosis. Data from three independent samples of non-clinical young adults were analyzed. Self-reports were administered to assess psychopathological symptoms (samples 1 – 3), neurodevelopmental risk factors for psychosis (sample 1), social defeat components (sample 2), childhood trauma and loneliness (sample 3). A total of 4782 individuals were surveyed across all three samples. Unadjusted analyses showed that the following factors are associated with higher odds of FP: obstetric complications, childhood trauma, non-right handedness, a lower education level, unemployment, minority status, humiliation, perceived constraints, and loneliness. However, only minority status and a lower level of education were associated with higher odds of FP after adjustment for other psychopathological symptoms, age, and gender. Importantly, hallucination-like experiences across all recorded modalities and paranoia were associated with higher odds of FP in all samples. Depressive symptoms were weakly associated with FP in two samples. Findings from the present study suggest that the majority of known risk factors for psychosis contribute to the emergence of FP through the effects on psychotic experiences. Low educational attainment and minority status might be the only risk factors independently contributing to the emergence of FP.

## 1 Introduction

The phenomenon of felt presence (FP) —the sensation of an unseen ‘other’ being near despite the absence of any direct sensory input—represents a fascinating yet underexplored frontier in psychiatric research. While FP is often linked to hallucinations and traditionally associated with neurological disorders, it is also regularly experienced as a normal and non-distressing phenomenon within the general population, particularly in situations of grief and bereavement (3). Despite being prevalent in both clinical settings and broader contexts, FP remains relatively unrecognized and poorly comprehended. Given the prevalence of FP in both clinical and non-clinical settings, it should not be confined to individuals with mental disorders. Instances of FP have been documented across various situations, including among the general population, participants in mountaineering expeditions (1), individuals experiencing bereavement or near-death situations (2, 3), episodes of sleep paralysis (4), as well as post-partum state (5), loneliness (6, 7), and poor sleep (7). Instead of being solely seen as abnormal or pathological, FP is often a typical part of human experience, especially in situations like grief, where it is commonly regarded as a reassuring and welcomed presence. Traditionally associated with neurological conditions like epileptic seizure (8), Parkinson’s disease (9) and psychiatric anomalies such as schizophrenia, FP offers a unique window into the interplay between psychopathology, neurobiology, and contextual background. It is important to note that FP is not merely a perceptual anomaly but can also be understood as an unusual yet meaningful experience that occurs in various psychological and cultural contexts, which poses significant questions about the underlying mechanisms of human consciousness and sensory perception.

Historically, studies on FP have varied widely in focus and scope. Nielsen’s exploration of the phenomenon during sleep paralysis suggests it may arise from dream-like cognitive processes rather than mere sensory misfiring (4). Barnby et al. (10) highlight that the characteristics of FP, such as the level of personification, frequency, intensity, distress, and meaning differ based on individual factors and the socio-cultural context in which they occur. For example, within religious environments, FP is typically seen as a positive and comforting presence, widely accepted among community members (11). Conversely, individuals at an ultra-high risk for psychosis tend to report FP with a greater frequency and vividness, often accompanied by significant distress (12). Barnby et al. (10) expanded on these observations by associating FP with a range of psychiatric symptoms, suggesting its manifestation across a continuum of psychological states from typical experiences to pathological conditions (10).

Despite this, the literature often lacks a unifying framework. For instance, the study by Alderson-Day et al. (13) examined FP within specific demographics, thus revealing its variable manifestation dependent on personal and cultural contexts. The authors employed a mixed-methods approach through three online surveys that explored the frequency, characteristics, and correlates of FP among the general population samples, individuals with spiritual beliefs, and those engaged in solo endurance activities. The findings suggest that FP experiences are common across different contexts and are strongly associated with the general hallucination proneness. However, nuances exist, with proneness to paranoia and male gender also playing significant roles, particularly in the general population where such experiences are more prevalent (13). Finally, two recent studies (6, 7) explored various aspects of FP and hallucinations in relation to social factors, such as loneliness, past occurrence of adverse events, and poor sleep. Findings from one of these studies revealed that increased loneliness correlates significantly with social hallucinations, where voices or touches are personified as coming from a social agent, but not with non-social hallucinations (e.g., hearing music), in a sample of young adults (6).. The second study found that 1.6% of the sample (165 individuals) experienced FP in the past month, while these experiences were often accompanied by visual and tactile hallucinations as well as delusion-like thinking. Notably, FP was associated with prior adverse events, loneliness, and poor sleep quality, suggesting that FP could serve as an indicator of the general hallucination proneness (7).

In sum, previous studies have variably interpreted FP as a byproduct of disrupted sensory processing, a manifestation of extreme psychological states, or a response to social isolation. Moreover, FP has often been conceptualized as a part of psychotic symptoms or at least a significant correlate of hallucination-like experiences. However, it appears to be largely unknown as to whether FP shares overlapping risk factors with hallucination-like experiences. However, FP should also be recognized as a normative experience, particularly within the context of grief, where it often plays a non-pathological role. Given previously noted associations, we sought to clarify whether FP is linked to commonly recognized psychosis risk factors across three independent community samples without prior exposure to psychiatric treatment. Specifically, in the present study, we tested for the relationship of FP with psychopathological symptoms, neurodevelopmental risk factors for psychosis, social defeat components, childhood trauma, and loneliness.

## 2 Material and methods

### 2.1 Participants

Participants were enrolled across three independent studies through an internet-based survey. The study was carried out by a research company using its own platform designed to perform internet-based surveys. In all of them, participants were considered eligible if they reported a negative lifetime history of psychiatric treatment. Additionally, participants were enrolled if they met the criterion of age, i.e., 18 – 35 years in study 1; 18 – 40 years in studies 2 and 3. The survey had an anonymous character and all participants were informed about its confidentiality. All of them agreed to participate in the survey. All studies received approval of the Bioethics Committee at Wroclaw Medical University, Wroclaw, Poland (approval numbers: 99/2023, 22/2024, and 240/2024).

### 2.2 Psychopathological symptoms

To measure hallucination-like experiences and FP, we used the Prodromal Questionnaire-16 (PQ-16) across all samples (14). It was designed in order to screen for psychosis risk states and includes 16 questions with yes-or-no responses addressing the presence of various hallucination-like experiences. In this study, we focused on preceding 4 weeks and analyzed the following phenomena: gustatory/olfactory hallucination-like experiences (item 3: “I sometimes smell or taste things that other people can’t smell or taste”), visual hallucination-like experiences (item 8: “I have seen things that other people apparently can’t see”), auditory hallucination-like experiences (item 4: “I often hear unusual sounds like banging, clicking, hissing, clapping or ringing in my ears”, item 12: “Sometimes I feel suddenly distracted by distant sounds that I am not normally aware of”, and item 13: “I have heard things other people can’t hear like voices of people whispering or talking”; the total score ranged between 0 and 3), and FP (item 15: “I have had the sense that some person or force is around me, even though I could not see anyone”).

Depressive symptoms were measured using the Patient Health Questionnaire-9 (PHQ-9) (15). The PHQ-9 refers to symptoms experienced over preceding 2 weeks. All items are rated on a 4-point scale with responses ranging from 0 – “not at all” to 3 – “nearly every day”. The total PHQ-9 score ranges between 0 and 27 (higher scores indicated higher levels of depressive symptoms).

Anxiety symptoms were assessed using the Generalized Anxiety Disoder-7 (GAD-7) (16). The GAD-7 refers to symptoms experienced over preceding 2 weeks. All items are rated on a 4-point scale (0 – “not at all”; 3 – “nearly every day”). The total PHQ-9 score ranges between 0 and 21 (higher scores indicated higher levels of anxiety symptoms).

### 2.3 Study-specific risk factors

A detailed description of measures used to assess exposure to specific risk factors is provided in **Supplementary Table 1**.

In brief, study 1 focused on recognizing the impact of risk factors for hallucination-like experiences operationalized within the exposome score. Specifically, self-reports were used to measure winter season of birth (17), obstetric complications, advanced paternal age (father’s age over > 35 years at the time of birth) (18), non-right handedness, urban upbringing, a history of childhood trauma, and problematic cannabis use. A history of childhood trauma (emotional neglect, emotional abuse, physical abuse, and sexual abuse) was assessed for adversities under the age of 18 years using selected items from the Traumatic Experience Checklist (TEC) (19) and the Childhood Experience of Care and Abuse Questionnaire (CECA.Q) (20, 21). In turn, to record problematic cannabis use, selected items from the Cannabis Problems Questionnaire (CPQ) were administered (22).

Study 2 was developed to understand the associations between social defeat components and hallucination-like experiences. As the measures of social defeat, the following constructs were assessed: (1) socioeconomic status (the level of education, employment status, and monthly income); (2) humiliation; (3) minority status; (4) perceived constraints and domain control (23, 24).

Study 3 included the measures of loneliness and a history of childhood trauma. The study was primarily designed to assess the association of these measures with various domains of psychopathology. To measure a history of childhood trauma, the same items as in study 1 were used. In turn, loneliness was assessed using the Revised UCLA Loneliness Scale (R-UCLA) (25, 26).

### 2.4 Data analysis

There were no missing data in this study. Differences across samples were assessed using the χ^2^ (categorical variables) and t-tests (continuous variables). Next, binary logistic regression analysis was used to test the association of risk factors (independent variables) with FP (dependent variable). This part of data analysis was carried out following a stepwise procedure. The first block of variables was limited to risk factors of FP. Next, the measures of psychopathology (depressive symptoms, anxiety symptoms, and hallucination-like experiences) were added to the model. Finally, results were adjusted for age and gender. The percentage of variance in FP explained by each model was assessed by estimating the Nagelkerke R^2^. Results were considered significant if the p-value was lower than 0.05. Data analysis was carried out in the SPSS software, version 28.

## 3 Results

### 3.1 The general characteristics across studies

**Figure 1:**
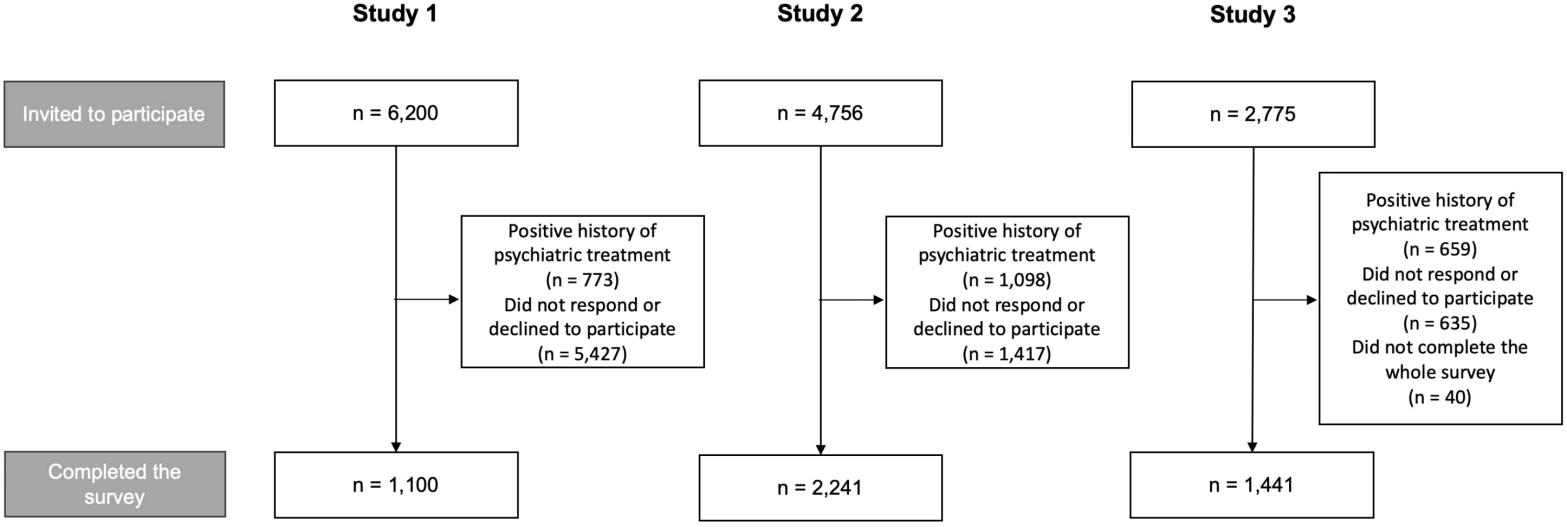
Study design.

The number of individuals invited to participate in each study was 1,100 in study 1 (51.4% females, aged 18 – 35 years), 2,241 in study 2 (53.4% females, aged 18 – 40 years), and 1441 (51.4% females, aged 18 – 40 years) in study 3 (**Figure 1**). As expected, participants from study 1 were significantly younger compared to those from studies 2 and 3, while differences in gender and education across studies were not significant (**Table 1**). The level of all categories of hallucination-like experiences, except for visual hallucinations, and depressive symptoms was significantly higher in participants from study 1 compared to those from studies 2 and 3. The same pattern was observed for the prevalence of self-reported FP. Also, the level of anxiety symptoms was significantly higher in participants from study 1 compared to those from study 3 as well as in participants from study 2 compared to those from study 3.

**Table 1:** General characteristics of the samples and their comparison.

	Study 1 (*n* = 1100)	Study 2 (*n* = 2241)	Study 3 (*n* = 1441)	p	Pairwise comparisons
Age, years	27.1 ± 5.1	30.3 ± 6.3	29.5 ± 6.3	**< 0.001**	1 < 2, 1 < 3
Gender, %F	565 (51.4)	1197 (53.4)	740 (51.4)	0.332	–
Education Primary Vocational Secondary Higher	61 (5.5) 89 (8.1) 553 (50.3) 397 (36.1)	64 (2.9) 161 (7.2) 908 (40.5) 1108 (49.4)	91 (6.3) 109 (7.6) 617 (42.8) 624 (43.3)	**< 0.001**	–
Felt presence	238 (21.6)	370 (16.5)	233 (16.2)	**< 0.001**	1 > 2, 1 > 3
Gustatory/olfactory hallucinations	427 (38.8)	682 (30.4)	467 (32.4)	**< 0.001**	1 > 2, 1 > 3
Auditory hallucinations	533 (48.5)	827 (36.9)	569 (39.5)	**< 0.001**	1 > 2, 1 > 3
Visual hallucinations	167 (15.2)	276 (12.3)	183 (12.7)	0.061	–
Paranoia	475 (43.2)	762 (34.0)	507 (35.2)	**< 0.001**	1 > 2, 1 > 3
Depressive symptoms	9.4 ± 6.2	8.1 ± 5.6	7.9 ± 5.6	**< 0.001**	1 > 2, 1 > 3
Anxiety symptoms	7.6 ± 5.5	7.4 ± 5.2	6.3 ± 5.2	**< 0.001**	1 > 3, 2 > 3

Significant differences (p < 0.05) are marked in bold.

Data provided as mean ± SD or n (%).

### 3.2 Factors associated with FP across studies

#### 3.2.1 Study 1

Unadjusted analysis (**Table 2**) revealed that a history of any childhood adversities (OR = 2.474, 95%CI: 1.529 – 4.002, p < 0.001), non-right handedness (OR = 1.762, 95%CI: 1.024 – 3.031, p = 0.041), and exposure to obstetric complications (OR = 1.466, 95%CI: 1.018 – 2.112, p = 0.040) are associated with the occurrence of FP. However, all of these associations appeared to be not significant after adding the measures of hallucination-like experiences, depressive, and anxiety symptoms to the model. When controlling for the effects of age and gender, the following measures of psychopathology were associated with the occurrence of FP: depressive symptoms (OR = 1.068, 95%CI: 1.012 – 1.127, p = 0.017), gustatory/olfactory hallucinations (OR = 2.356, 95%CI: 1.510 – 3.676, p < 0.001), visual hallucinations (OR = 3.095, 95%CI: 1.826 – 5.246, p < 0.001), paranoia (OR = 4.133, 95%CI: 2.564 – 6.662, p < 0.001), and auditory hallucinations (OR = 1.912, 95%CI: 1.513 – 2.415, p < 0.001).

**Table 2:** Results of binary logistic regression analysis for study 1 showing the association of psychopathological symptoms and exposome components with felt presence.

Model	Independent variable	OR	95%CI	p
Model 1 (exposome components), R^2^ = 0.305	Winter season of birth	0.976	0.644 – 1.479	0.909
	Obstetric complications	1.466	1.018 – 2.112	**0.040**
	Advanced paternal age	1.088	0.731 – 1.620	0.678
	Non-right handedness	1.762	1.024 – 3.031	**0.041**
	Childhood trauma	2.474	1.529 – 4.002	**< 0.001**
	Problematic cannabis use	1.131	0.992 – 1.289	0.066
	Urbanicity	0.900	0.627 – 1.290	0.900
Model 2 (exposome components and psychopathology), R^2^ = 0.436	Winter season of birth	1.106	0.674 – 1.816	0.690
	Obstetric complications	1.154	0.739 – 1.802	0.528
	Advanced paternal age	0.840	0.510 – 1.385	0.840
	Non-right handedness	1.649	0.838 – 3.244	0.147
	Childhood trauma	1.176	0.639 – 2.165	0.602
	Problematic cannabis use	1.008	0.859 – 1.183	0.920
	Urbanicity	0.838	0.537 – 1.308	0.437
	Anxiety symptoms	0.954	0.897 – 1.014	0.132
	Depressive symptoms	1.068	1.012 – 1.127	**0.017**
	Gustatory/olfactory hallucinations	2.362	1.516 – 3.679	**< 0.001**
	Visual hallucinations	3.152	1.869 – 5.315	**< 0.001**
	Paranoia	4.096	2.548 – 6.585	**< 0.001**
	Auditory hallucinations	1.916	1.517 – 2.419	**< 0.001**
Model 3 (exposome components, psychopathology, age, and gender), R^2^ = 0.436	Winter season of birth	1.110	0.675 – 1.826	0.681
	Obstetric complications	1.144	0.731 – 1.789	0.557
	Advanced paternal age	0.833	0.503 – 1.378	0.477
	Non-right handedness	1.641	0.833 – 3.232	0.152
	Childhood trauma	1.176	0.639 – 2.166	0.602
	Problematic cannabis use	1.002	0.853 – 1.177	0.977
	Urbanicity	0.821	0.522 – 1.292	0.394
	Anxiety symptoms	0.956	0.898 – 1.017	0.956
	Depressive symptoms	1.068	1.012 – 1.127	**0.017**
	Gustatory/olfactory hallucinations	2.356	1.510 – 3.676	**< 0.001**
	Visual hallucinations	3.095	1.826 – 5.246	**< 0.001**
	Paranoia	4.133	2.564 – 6.662	**< 0.001**
	Auditory hallucinations	1.912	1.513 – 2.415	**< 0.001**
	Age	1.000	0.958 – 1.043	0.986
	Gender, female	0.893	0.564 – 1.415	0.630

Significant associations (p < 0.05) are marked in bold.

#### 3.2.2 Study 2

Unadjusted analysis (**Table 3**) demonstrated that a lower level of education (OR = 0.669, 95%CI: 0.563 – 0.793, p < 0.001), unemployment status (OR = 1.586, 95%CI: 1.011 – 2.489, p = 0.025), minority status (OR = 1.761, 95%CI: 1.349 – 2.298, p < 0.001), humiliation (OR = 1.036, 95%CI: 1.026 – 1.047, p < 0.001), and perceived constraints (OR = 0.987, 95%CI: 0.975 – 1.000, p = 0.045) are significantly associated with the occurrence of FP. However, except for minority status (OR = 1.445, 95%CI: 1.040 – 2.008, p = 0.028) none of these factors appeared to be associated with FP after adjustment for the measures of psychopathology, age, and gender. Simultaneously, there were significant associations of depressive symptoms (OR = 1.047, 95%CI: 1.000 – 1.095, p = 0.048), gustatory/olfactory hallucinations (OR = 2.419, 95%CI: 1.776 – 3.296, p < 0.001), visual hallucinations (OR = 3.546, 95%CI: 2.410 – 5.218, p < 0.001), paranoia (OR = 1.913, 95%CI: 1.367 – 2.677, p < 0.001), and auditory hallucinations (OR = 2.367, 95%CI: 1.997 – 2.804, p < 0.001) with FP after adjustment for age and gender.

**Table 3:** Results of binary logistic regression analysis for study 2 showing the association of psychopathological symptoms and social defeat components with felt presence.

Model	Independent variable	OR	95%CI	p
Model 1 (social defeat components), R^2^ = 0.105	Education, higher	0.669	0.563 – 0.793	**< 0.001**
	Unemployed	1.586	1.011 – 2.489	**0.045**
	Monthly income	1.017	0.857 – 1.208	0.847
	Minority status	1.761	1.349 – 2.298	**< 0.001**
	Humiliation	1.036	1.026 – 1.047	**< 0.001**
	Perceived constraints	0.987	0.975 – 1.000	**0.045**
	Domain control	0.993	0.979 – 1.008	0.370
Model 2 (social defeat components and psychopathology), R^2^ = 0.441	Education, higher	0.688	0.558 – 0.848	**< 0.001**
	Unemployed	1.579	0.922 – 2.705	0.096
	Monthly income	0.996	0.805 – 1.233	0.973
	Minority status	1.425	1.028 – 1.975	**0.034**
	Humiliation	1.000	0.987 – 1.014	0.954
	Perceived constraints	1.000	0.984 – 1.017	0.998
	Domain control	1.000	0.981 – 1.018	0.978
	Depressive symptoms	1.045	0.999 – 1.093	0.057
	Anxiety symptoms	0.985	0.938 – 1.035	0.559
	Gustatory/olfactory hallucinations	2.396	1.761 – 3.261	**< 0.001**
	Visual hallucinations	3.610	2.459 – 5.299	**< 0.001**
	Paranoia	1.930	1.381 – 2.699	**< 0.001**
	Auditory hallucinations	2.362	1.993 – 2.798	**< 0.001**
Model 3 (social defeat components, psychopathology, age, and gender), R^2^ = 0.442	Education, higher	0.683	0.551 – 0.846	**< 0.001**
	Unemployed	1.578	0.915 – 2.723	0.101
	Monthly income	0.966	0.773 – 1.207	0.760
	Minority status	1.445	1.040 – 2.008	**0.028**
	Humiliation	1.000	0.987 – 1.014	0.965
	Perceived constraints	1.000	0.984 – 1.017	0.967
	Domain control	1.000	0.982 – 1.019	0.977
	Depressive symptoms	1.047	1.000 – 1.095	**0.048**
	Anxiety symptoms	0.986	0.939 – 1.036	0.583
	Gustatory/olfactory hallucinations	2.419	1.776 – 3.296	**< 0.001**
	Visual hallucinations	3.546	2.410 – 5.218	**< 0.001**
	Paranoia	1.913	1.367 – 2.677	**< 0.001**
	Auditory hallucinations	2.367	1.997 – 2.804	**< 0.001**
	Age	1.010	0.984 – 1.036	0.456
	Gender, male	0.895	0.644 – 1.244	0.509

Significant associations (p < 0.05) are marked in bold.

R^2^ refers to Nagelkerke R^2^.

#### 3.2.3 Study 3

Unadjusted analysis (**Table 4**) showed that both loneliness (OR = 1.033, 95%CI: 1.018 – 1.047, p < 0.001) and a history of any childhood trauma (OR = 1.906, 95%CI: 1.324 – 2.744, p < 0.001) are significantly associated with FP. However, after adding the effects of hallucination-like experiences, depressive symptoms, and anxiety symptoms, none of these factors appeared to be significantly associated with FP. Simultaneously, gustatory/olfactory hallucinations (OR = 2.020, 95%CI: 1.435 – 2.844, p < 0.001), visual hallucinations (OR = 2.338, 95%CI: 1.548 – 3.529, p < 0.001), paranoia (OR = 1.748, 95%CI: 1.205 – 2.534, p = 0.003), and auditory hallucinations (OR = 2.079, 95%CI: 1.724 – 2.505, p < 0.001) were associated with FP after adjustment for age and gender.

**Table 4:** Results of binary logistic regression analysis for study 3 showing the association of psychopathological symptoms, childhood trauma history, and loneliness with felt presence.

Model	Independent variable	OR	95%CI	p
Model 1 (loneliness and childhood trauma), R^2^ = 0.055	Loneliness	1.033	1.018 – 1.047	**< 0.001**
	Childhood trauma	1.906	1.324 – 2.744	< 0.001
Model 2 (loneliness, childhood trauma, and psychopathological symptoms), R^2^ = 0.324	Loneliness	0.997	0.979 – 1.016	0.771
	Childhood trauma	1.141	0.753 – 1.729	0.533
	Depressive symptoms	1.010	0.964 – 1.059	0.671
	Anxiety symptoms	1.039	0.989 – 1.092	0.127
	Gustatory/olfactory hallucinations	2.015	1.433 – 2.835	**< 0.001**
	Visual hallucinations	2.294	1.526 – 3.448	**< 0.001**
	Paranoia	1.716	1.186 – 2.482	**< 0.001**
	Auditory hallucinations	2.043	1.698 – 2.460	**< 0.001**
Model 3 (loneliness, childhood trauma, psychopathological symptoms, age, and gender), R^2^ = 0.327	Loneliness	0.996	0.978 – 1.015	0.677
	Childhood trauma	1.155	0.760 – 1.754	0.500
	Depressive symptoms	1.018	0.970 – 1.067	0.474
	Anxiety symptoms	1.016	0.985 – 1.089	0.167
	Gustatory/olfactory hallucinations	2.020	1.435 – 2.844	**< 0.001**
	Visual hallucinations	2.338	1.548 – 3.529	**< 0.001**
	Paranoia	1.748	1.205 – 2.534	**0.003**
	Auditory hallucinations	2.079	1.724 – 2.505	**< 0.001**
	Age	1.024	0.997 – 1.051	0.084
	Gender, male	1.069	0.762 – 1.501	0.698

Significant associations (p < 0.05) were marked in bold.

R^2^ refers to Nagelkerke R^2^.

## 4 Discussion

Findings from the present study revealed that the association of known risk factors for psychosis, except for a minority status and low educational attainment, with FP appears to be not significant after adjustment for hallucination-like experiences, depressive symptoms, and anxiety symptoms. Simultaneously, all categories of hallucination-like experiences were found to be associated with the occurrence of FP. With respect to non-psychotic psychopathology, we found that depressive symptoms, but not anxiety symptoms, might be weakly associated with FP (evidence supported by findings from two, out of three tested samples).

The exact mechanisms underlying the association of minority status with FP after adjustment for other psychopathological symptoms remain unknown. One of potential explanations is that FP in people representing social minorities might be the effect of social isolation. Indeed, individuals with minority status might show difficulties in social integration (27). Although we did not observe a significant association of loneliness with FP after controlling for other psychopathological symptoms, it is needed to note that social isolation and loneliness represent different constructs. Indeed, loneliness is a subjective phenomenon that can be defined as a discordance between actual and desired relationships (28). To support, this perspective, it is needed to note that FP has been conceptualized as the phenomenon that protects from social isolation (29). It has been reported in relatives of deceased individuals as the way of coping with an objective loss within a close environment (2, 30). The social deafferentation hypothesis (31), although developed on the ground of psychosis research, points to these considerations. According to this theory, hallucination-like experiences often have a social context that appears to be either familiar or distressing for an individual. Apart from the social isolation perspective, it is needed to note that minority status is often related to stressful experiences (32, 33). Observations from our study suggest that FP is associated with all categories of hallucination-like experiences including paranoia, gustatory, olfactory, auditory, and visual hallucinations. This is in line with results of the previous study (7) based on a large population sample, which showed that people who experienced FP present a higher prevalence of visual and tactile hallucinations in the preceding month, as well as delusion-like thinking in comparison with people without FP experiences. However, some studies have not reported these observations consistently. For instance, Larøi et al. (34) did not find a significant association of FP with auditory hallucinations, whereas they determined that FP is more frequently observed among individuals experiencing tactile and olfactory hallucinations. Recent findings from the study by Alderson-Day et al. (13) based on two samples of people with spiritual and spiritualist beliefs and practitioners of endurance/solo pursuits showed that in both samples FP was significantly associated with auditory and visual hallucinations, while there was no significant association with paranoia. It is important to note that this study was based on relatively small, specifically selected samples and that participants were much older than participants form our study.

It is also necessary to discuss the prevalence of FP in our sample that varied between 16.2% (study 3) and 21.6% (study 1). It should be noted that the samples differed significantly in terms of age, with significantly younger participants in sample 1 compared to samples 2 and 3. Previous studies have reported that the prevalence of hallucination-like experiences decreases with age (35–38). Given that FP is an important correlate of hallucination-like experiences, its prevalence should also decrease with age. However, studies reporting the prevalence of FP have provided mixed findings. For instance, in the study performed in the Netherlands, the prevalence rate reached 3.1% in the preceding month among participants aged 14 – 88 years (36). In another study (34), based on a large, randomly selected and representative sample of the Norwegian population, there was a significant difference in the prevalence of FP was observed. It reached 12.3% in people aged 19 – 30 years and 29.8% in those aged 61–96 years.

## 5 Limitations

There are certain limitations of the present study that require further discussion. It is important to keep in mind that this study was conducted in a non-clinical population, without in-person assessment of psychopathology using validated diagnostic instruments. Moreover, the use of the PQ-16 may result in the misclassification of non-pathological experiences, such as those related to grief or near-death phenomena, as psychotic symptoms, potentially skewing the interpretation of our findings. Therefore, caution should be taken when generalizing the results to clinical populations.

Also, various limitations related to the snowball method and sampling accuracy should be taken into consideration including those related to data accuracy and sample representativeness (39). Another limitation is that only one item was used to measure FP. At this point, it should be noted that some authors notice the heterogeneity of FP experiences in terms of the presence of underlying sensory content or attributed localization in some individuals (40). Moreover, contextual factors contributing to FP and sociocultural backgrounds of participants were not thoroughly assessed. Finally, conclusions about causality cannot be drawn due to a cross-sectional design.

## 6 Conclusions

In conclusion, findings from the present study suggest that the majority of known risk factors for psychosis contribute to the emergence of FP through the effects on hallucination-like experiences. Low educational attainment and minority status are the only factors that are associated with FP regardless of their associations with hallucination-like experiences. However, further studies are also needed to address limitations of the present study and investigate other mechanisms that might independently lead to the occurrence of FP. Clinical implications from the present study are also important and suggest that interventions focused on underlying hallucination-like experiences might lower the levels of FP, when distressing for the experiencer.

## Data Availability

The raw data supporting the conclusions of this article will be made available by the authors, without undue reservation.
