# Supplementary material for: Felt presence and its determinants in young adults: results from three independent samples
Source: Front Psychiatry. 2024 Sep 13;15:1442313. doi: 10.3389/fpsyt.2024.1442313 (PMC11427248; doi:10.3389/fpsyt.2024.1442313)
Supplement: Supplementary file 1 [file Table1.docx]

**Supplementary Appendix**

**Supplementary Table 1.** Study-specific measures of risk factors for felt presence.

| Category of risk factors | Risk factors | Study | Description |
| --- | --- | --- | --- |
| Neurodevelopmental risk factors | Winter season of birth | 1 | Participants were asked to provide their month of birth. According to the meteorological seasons in the Northern Hemisphere, those born between December and February were assigned to the winter season, according to a recent meta-analysis by Coury et al. (Coury et al., 2023). |
|  | Obstetric complications | 1 | Exposure to obstetric complications was assessed using three items: “Were you delivered by caesarean section due to perinatal complications?”, “Was your birth weight less than 2500 g?”, and “Were you delivered preterm, i.e., before the 37th week of pregnancy?”. Possible responses were “yes”, “no”, and “I don’t know”. Participants were coded as those with a history exposure to obstetric complications if their response to any of these questions was “yes”. Responses of “I don’t know” were included as missing data. |
|  | Advanced paternal age | 1 | Participants were requested to provide the age of their father at the time of their birth. Advanced paternal age was defined as ≥ 35 years (Torrey et al., 2009). |
|  | Non-right handedness | 1 | Respondents were asked to provide their hand preference, i.e., whether they are right-handed, left-handed, or have mixed-handedness. In agreement with a prior meta-analysis, responses indicating left-handedness and mixed-handedness were included as a non-right-handedness. |
|  | Childhood trauma | 1, 3 | A total of six items were used to record a history of emotional neglect, emotional abuse, bullying, and sexual abuse before the age of 18. Among them the following items were derived from the Traumatic Experience Checklist (TEC) (Nijenhuis et al., 2002) to assess emotional neglect, abuse, and bullying: “When you were a child or a teenager, have you ever felt emotionally neglected (e.g., being left alone, insufficient affection) by your parents, brothers or sisters?”; “When you were a child or a teenager have you ever felt emotionally abused (e.g., being belittled, teased, called  names, threatened verbally, or unjustly punished) by your parents, brothers or sisters?”, and “When you were a child or teenager, did you experience psychological violence (e.g., nicknames, teasing) or physical abuse (e.g., jerking, beating) from your peers?”. In turn, exposure to childhood sexual abuse was recorded using items from the Childhood Experience of Care and Abuse (CECA.Q) (Bifulco et al., 2005; Kasznia et al., 2022): “When you were a child or teenager did you have any unwanted sexual experiences?”; “Did anyone force you or persuade you to have sexual intercourse against your wishes before age 17?” and “Can you think of any upsetting sexual experiences before age 17 with a related adult or someone in authority e.g., teacher?”. Participants who confirmed a history of any of these experiences were  classified as having been exposed to sexual abuse. Next, a history of any of these adversities was coded as a history of childhood trauma. |
|  | Urban upbringing | 1 | Participants were asked to classify their primary residence across the following categories (1) rural; (2) a city of up to 100,000 inhabitants; (3) a city of 200,000–500,000 inhabitants; and (4) a city over 500,000 inhabitants. In the data analysis process, the responses were categorized into those reporting a rural or urban place of residence. |
| Social defeat | Education | 2 | Participants were requested to categorize their highest level of education across the following categories: (1) primary; (2) vocational; (3) secondary and (4) higher. Next, the level of education was coded as an ordinal variable. |
|  | Employment status | 2 | Participants were requested to categorize their current employment status according to the following classification: (1) unemployment; (2) part-time employment; (3) full-time employment and (4) student status. Next, they were categorized as employed or students vs. unemployed. |
|  | Monthly income | 2 | Monthly income was categorized according to the following equivalents: (1) < 750 USD; (2) 750 – 1,500 USD; (3) 1,500 – 2,500 USD; (4) 2,500 – 3,750 USD and (5) > 3,750 USD. Subsequently, monthly income was included as an ordinal variable. |
|  | Minority status | 2 | Minority status was assessed using the following item: “Do you feel a part of the minority: (1) sexual minority; (2) religious minority; (3) cultural minority and (4) none?” Next, participants’ responses were dichotomized into the presence vs. absence of self-reported minority status. |
|  | Humiliation | 2 | The Humiliation Inventory was administered to assess internal experience of humiliation (Hartling and Luchetta, 1999). The questionnaire consists of 32 self-reported items rated between 1 ("not harmed at all") to 5 ("extremely harmed"). The questionnaire includes two subscales, i.e., the cumulative humiliation subscale and the fear of humiliation subscale. The first one measures the severity of past humiliating experiences, while the latter one records the level of anticipation and anxiety regarding anticipated humiliating experiences. In this study, the first subscale that based on 12 items was used. The total score ranges between 12 and 60, where higher scores correspond with higher levels of humiliation experiences. The Cronbach’s alpha was 0.961 in the present study. |
|  | Perceived constraints and domain control | 2 | The measures developed by Seeman et al. (2014), which build upon the concepts initially described by Lachman and Weaver Lachman and Weaver (1998), were used to assess individual sense of control in the social environment. The first one was the Perceived Constraints Scale (8 items) with each item rated on a 7-point scale (from 1 - "strongly agree" to 7 - "strongly disagree"). The second one was the Domain of Control Scale that includes 5 items rated on a 10-point scale (from 1 - "total lack of control" to 10 - "complete control"). The Cronbach’s alpha for Perceived Constraints Scale and Domain of Control Scale were 0.893 and 0.815, respectively. |
| Other factors | Problematic cannabis use | 1 | To assess problematic cannabis use, we used 11 out of 16 items from the Cannabis Problems Questionnaire (CPQ) (Copeland et al., 2005), which cover the period of preceding 12 months: “Have you tended to smoke more on your own than you used to?”; “Have you been neglecting yourself physically?”; “Have you felt depressed for more than a week?”; “Have you been so depressed you felt like doing away with yourself?”; “Have you given up recreational activities you once enjoyed for smoking?”; “Do you find it hard to get the same enjoyment from your usual interests?”; “Have you felt more antisocial after smoking?”; “Have you worried about getting out of touch with friends or family?”; “Have you been concerned about a lack of motivation?”; “Have you worried about feelings of personal isolation or detachment?” and “Do you usually have a smoke in the morning, to get yourself going?”. Problematic cannabis use was coded as the number of positive responses across these items. |
|  | Loneliness | 3 | The Revised UCLA Loneliness Scale (R-UCLA) was used to measure the level of loneliness (Russell et al., 1980; Kwiatkowska et al., 2018). It is based on 20 items rated on a 4-point scale (from 1 – “never” to 4 – “often”). The total score ranges between 20 and 80 with higher scores indicating higher levels of loneliness. In the present study, the Cronbach’s alpha of the R-UCLA was 0.911. |

**References**

Bifulco, A., Bernazzani, O., Moran, P. M., Jacobs, C., 2005. The childhood experience of care and abuse questionnaire (CECA.Q): Validation in a community series. Br J Health Psychol 44(4), 563–581.

Copeland, J., Gilmour, S., Gates, P., Swift, W., 2005. The Cannabis Problems Questionnaire: factor structure, reliability, and validity. Drug Alcohol Depend 80(3), 313–319.

Coury, S. M., Lombroso, A., Avila-Quintero, V. J., Taylor, J. H., Flores, J. M., Szejko, N., Bloch, M. H., 2023. Systematic review and meta-analysis: Season of birth and schizophrenia risk. Schizophr Res 252, 244–252.

Hartling, L. M., Luchetta, T., 1999. Humiliation: Assessing the impact of derision, degradation, and debasement. J Prim Prev 19(4), 259–278.

Kasznia, J., Stańczykiewicz, B., Pytel, A., Szczygieł, K., Misiak, B., 2022. Psychometric properties of the Childhood Experience of Care and Abuse Questionnaire (CECA.Q) in a sample of individuals with schizophrenia from Poland. Arch Psychiatry Psychother 24(1), 42-48.

Kwiatkowska, M. M., Rogoza, R., Kwiatkowska, K., 2018. Analysis of the psychometric properties of the Revised UCLA Loneliness Scale in a Polish adolescent sample. Curr Issues Pers Psychol 6(2), 164-170.

Lachman, M. E., Weaver, S. L., 1998. The sense of control as a moderator of social class differences in health and well-being. J Pers Soc Psychol 74(3), 763–773.

Nijenhuis, E. R. S., Van der Hart, O., Kruger, K., 2002. The psychometric characteristics of the Traumatic Experiences Checklist (TEC): First findings among psychiatric outpatients. Clin Psychol Psychother 9(3), 200–210.

Russell, D. W., Peplau, L. A., Cutrona, C. E., 1980. The Revised UCLA Loneliness Scale: Concurrent and discriminant validity evidence. J Pers Soc Psychol 39, 472–480.

Seeman, M., Stein Merkin, S., Karlamangla, A., Koretz, B., Seeman, T., 2014. Social status and biological dysregulation: the "status syndrome" and allostatic load. Soc Sci Med 118, 143–151.

Torrey, E. F., Buka, S., Cannon, T. D., Goldstein, J. M., Seidman, L. J., Liu, T., Hadley, T., Rosso, I. M., Bearden, C., Yolken, R. H., 2009. Paternal age as a risk factor for schizophrenia: how important is it?. Schizophr Res 114(1-3), 1–5.
